# 

**Published:** 1888-08-04

**Authors:** 


### "THE HOSPITAL" LIBRARY OF NEW AND STANDARD WORKS.

For all information respecting the appearance of Titles, Prices, etc., of Books in this page, apply to Mr. W. H. HOWE, office of THE HOSPITAL, 38, Old Jewry.

11th edition, 2s, post free for 26 stamps.

STAMMERING, STUTTERING, LISPING, &c.; their Causes and Cure. By W. ABBOTTS, M D., Physician to the Hospital for Speech Affections.

BEAUMONT and Co., 6, Wells Street, Oxford Street, W. (72)

Crown 8vo., cloth, 2s 6d.

THE COMMONER DISEASES AND ACCIDENTS TO LIFE AND LIMB Firstly, their Prevention; secondly, their Immediate Treatment. By M. M. BASIL, M.A., M.B., C.M. (Edin.).

"Prief, clear, and good."—*Athenæum.*

"Thoroughly readable."—*Hospital.*

London: J. and A. CHURCHILL, 11, New Burlington Street.

### Medical Library.

ABDOMEN: Diseases of the. By S. O. Habershon. 21/- *Churchill.*

CANCER: Its Allies, and other Tumours. By F. A. Purcell 10/6 *Churchill.*

DIPHTHERIA: Its Nature and Treatment. By Sir Morell Mackenzie. 5/- *Churchill.*

EAR: The Diseases of the. By A Hartmann. 9/- *Pentland.*

EYE: On Diseases of the. By C. B. Taylor. *Redway.*

HEART: A Practical Treatise on Diseases of the. By W. H. Walshe. 16/- *Smith and Elder.*

HELPS to Health. By Hy. C. Burdett. 1/6 *Kegan Paul.*

HELP in Sickness. By Hy. C. Burdett. 1/6 *Kegan Paul.*

LIVER: Diseases of the. By Geo. Harley. 21/- *Churchill.*

LUNGS: A Practical Treatise on Diseases of the. By W H. Walshe. 16/- *Smith and Elder.*

[illegible]WIFERY FOR MIDWIVES. A Manual of. By Fancourt [illegible]Barnes. 6/- *Smith and Elder.*

NERVOUS SYSTEM; A Manual of Diseases of the. By W. R. Gowers. 12/6 *Churchill.*

### Library of Biography and History.

BACON. By R. W. Church (Dean of St. Paul's). 5/- *Macmillan*

GLADSTONE; Anecdotes of. By an Oxford Man. 1/- *Hamilton.*

GREECE: A History of. Part I. By Edwin Abbott. 10/6. *Rivingtons.*

HENRY II. By Mrs J. R. Green. 2/6. *Macmillan.*

MITCHELL: Life of John. By Wm. Dillon. 2 vols. 21/- *Kegan Paul.*

### Library of Christian Literature.

A CENTURY OF CHRISTIAN PROGRESS AND ITS LESSONS. By J. Johnston. 3/- *Nisbet*

FAITHFUL DEPARTED: The Present State of the. By Rev. Geo. Body. 1/- *Masters.*

ST. JOHN, THE AUTHOR OF THE FOURTH GOSPEL. By H. H. Evans. 6/- *Nisbet.*

THE VICTORY OF THE CROSS. By B. F. Westcott. 3/6 *Macmillan.*

### Dictionaries and Encyclopædias.

EVERYBODY'S POCKET CYCLOPÆDIA. By E. Sheldon. 6d. *Saxon and Co.*

MIDDLE ENGLISH: A Concise Dictionary of. By A. L. Mayhew—W. W. Skeat. 7/6 *Froude.*

NATIONAL BIOGRAPHY: Dictionary of. By Leslie Stephens 15/- *Smith and Elder.*

NEW ENGLISH DICTIONARY ON HISTORICAL PRINCIPLES. Edited by James Murray. Part 4 (Bra-Cass). 12/6 *Froude.*

### Library of Fiction.

A BITTER REPENTANCE. By Lady Virginia Sandars. 3 vols. *Hurst and Blackett.*

A WOMAN'S FACE. By F. Warden. 3 vols. *Ward and Downey.*

CHILDREN OF GIBEON. By Walter Besant. 2/- *Chatto and Windus.*

EVE. A Romance. By Author of "John Herring." 2 vols. *Chatto and Windus.*

JESS. By H. Rider Haggard 2/6 *Smith and Elder.*

### To Secretaries and Matrons.

THE Editor will be glad to have a copy of the Regulations, and also of every Report issued from the Press of Asylums, Hospitals, Convalescent and Nursing Institutions, as well as those of other Charities, so soon as they are published, and an early copy in duplicate of all appeals for funds. Notices of Annual and other Meetings, Festival Dinners, etc., will be welcomed. All such communications should be addressed to *The Editor, The Lodge, Porchester Square, London, W.* Any superintendent, secretary, matron, or other chief official who will regularly send such documents and information will be registered to receive free of cost a cover for binding THE HOSPITAL in half-yearly volumes on sending name and address to the publisher.

An old writer says: "A long chin declareth a man to be peaceable, yet a babbler. They that have little chins are much to be avoided and taken heed of, for they are full of impiety and wickedness, and are spies like unto serpents. If the end of the chin be round, it is a sign of nice manners; but the chin of a real man is square.

"It often happens that mere activity is a waste of time, that people that have a morbid habit of being busy are often terrible time-wasters, while, on the contrary, those who are judiciously deliberate, and allow themselves intervals of leisure, see the way before them in those intervals, and save time by the accuracy of their calculation."—*P. G. Hamerton.*

### Amusements with Profit for all Ages.

**Rules.**—All answers must be addressed to the Prize Editor, The Hospital, 38, Old Jewry, Cheapside, London, E.C., not later than the *first* post on Thursday next. The full name and address must in all cases be given, but a *nom de plume* may be added for publication. Only one side f the paper must be written on.

### FOR MEN AND WOMEN.

### First Quarterly Poetical Competition.

Commenced May 26th, 1888; ends August 25th, 1888.

This quarter a PRIZE of ONE GUINEA will be given to the competitor who gains the highest number of marks for original poems, on any subjects suitable for insertion in this paper. All contributions sent in will be credited according to merit, eighteen marks being the highest score for any one contribution. This competition to alternate weekly with the set acrostic competition.

### Acrostic Competition.

This quarter the original acrostic competition will be discontinued, and A PRIZE of HALF-A-GUINEA will be given to the competitor who gains the highest score for solution of acrostics set. *One mark* only will be given to the competitors who solve all the lights of each acrostic.

Exception will be made in the case of *quotation acrostics*, when *two marks* will be given, *one* for the correct solution of all *lights*, and *one* mark for the *source* of the quotations.

This week credit will be given for

### No. 5.—Double Acrostic.

Armed for the strife, see the glad rustics go,
To wield us stoutly till the ranks lie low.

1. A trial some men meet with dread profound,
And some submitting, have a blessing found.

2. A graceful order, used by not a few,
Who neither name nor origin e'er knew.

3. If you are prudent shun me, or you may
Remain in my dark limbo many a day.

4 The Highlander regards with clannish pride
That which the Saxon ventures to deride.

5 An odious reptile which we shuddering see,
And yet in need we gladly to it flee.

6 Be moderate if you would find content,
Shun meanness, but let money be well spent.

### Answers.

No. 4. Double Acrostic.

(Southey).

| | | | |
|---|---|---|---|
| L | ancelo | T | Tennyson, Idylls of the King. |
| A | lbert Graem | E | Scott, Lay of the Last Minstrel. |
| U | rie | N | Gray, Bard. |
| R | ega | N | Shakespeare, King Lear. |
| E | mil | Y | Wordsworth, White Doe. |
| A | donai | S | Shelley, Adonais. |
| T | asso | O | Byron, Childe Harold. |
| E | inio | N | Scott, Lord of the Isles. |

Several answers have been received to the above acrostic, but no competitor has succeeded in solving all the lights correctly.

### THE WORD COMPETITION.

Third Quarterly Competition commenced

May 5th, 1888; ends August 4th, 1888.

Three prizes of one guinea, 15*s.* and 10*s.* 6*d.* will be given each quarter to the three competitors who obtain the highest number of marks by making the largest number of words derived from the word or phrase set for anatomical dissection; that for this, the *thirteenth* week of the quarter, being

"Holidays."

Observe.—Proper names, abbreviations, foreign words, words of less than four letters, and repetitions are barred; plurals, and past and present participles of verbs are allowed. Nuttall's *Standard* dictionary only to be used.

N.B.—All words sent in must be arranged *alphabetically*, with correct total affixed.

### Results of Eleventh Week Puzzles.

*Italian.*—Tinie (13) Patience (12), Dodo (12), Sym (11), Reldas (11), Lightowlers (10), Lauriston (10), Alicia (9), Puff (8).

Tenth Week.—Tinie (30).

### THE CHILDREN'S CORNER.

PRIZES.

FOR MOST CORRECT ANSWERS TO PUZZLES.

This Commenced October 1st, 1887.

One Pound a Month.

A PRIZE OF THREE GUINEAS

to the Competitor who shall have sent in the largest number of correct or best answers during the twelve months.

### First Week Puzzles.

No. 1. Enigma.

There is a shoemaker who works without leather,
But calls to his aid the elements together;
Of fire he makes use, water, earth, air,
And for every customer makes a double pair.

No. 2. Geographical Diamond Puzzle

| | |
|---|---|
| a | 1. A consonant. |
| a e i | 2. An animal, |
| i n n n p | 3. A country. |
| p p s | 4. A useful article. |
| s | 5. A consonant. |

No. 3. Biographical Acrostic.—The initials read downwards and the finals upwards will give the names of two brave defenders. 1. A famous English General. 2. One of Shakespeare's Plays. 3. A French author. 4. A measure. 5. A bird. 6 A nautical narrative.

No. 4. Birds Enigmatically Expressed.—1. A plaything. 2. An instrument to raise weights. 3. A sound indicative of triumph.

### Results of Third Week Puzzles.

No 9 Historical Charade—Bannock-burn=Bannockburn.

No. 10. Double Acrostic.

| | | |
|---|---|---|
| C | uf | F |
| O | ctobe | R |
| L | av | A |
| U | r | N |
| M | ee | K |
| B | il | L |
| U | r | I |
| S | overeig | N |

No. 11. Decapitation.—Shark. Hark. Ark.

No. 12. Charade.—Cock-roach=Cockroach.

| Marks | July 14th. | Total of First, Second, and Third Weeks. |
|---|---|---|
| A. C. K. | 4 | 11 |
| Alsie | 3 | 8 |
| Boadicea | — | 7 |
| *Curly | 3 | 10 |
| Happy Eliza | 3 | 9 |
| Hercules | 3 | 9 |
| Jack | 3 | 10 |
| *Geranium | 4 | 10 |
| Poodle | 3 | 8 |
| Thanet | 3 | 10 |
| Scrooge | 4 | 12 |
| Young Surgeon | 3 | 9 |
| Gem | 3 | 7 |
| W. R. M. | 3 | 3 |
| Judy | 3 | 7 |

### Conditions.

I. Every paper sent in must be clearly written, and one side only must be used. All competitors must mark at the head of their papers the number of their competition.

II. Each paper must be signed by the author with his or her real name and address. A *nom de plume* may be added, if the writer does not desire to be referred to by us by his real name. In the case of all prizewinners, however, the real name and address will be published.

III. All communications relating to prize competitions should be addressed to "The Prize Editor, The Hospital, 38, Old Jewry, London, E.C." Competitors are requested not to include in letters, etc., to the Prize Editor communications on matters of other business. All letters must be fully prepaid.

IV. The Prize Editor of The Hospital is the sole judge of the competitions, and his decision is in all cases final and without appeal.

V. The Prize Editor reserves the right to publish any paper sent in, whether it receives a prize or not. He does not hold himself responsible for any MSS. sent, nor can he undertake to return rejected competitions.

VI.—All children resident at school are requested to forward their *home* address in addition to the school one, when entering for the "Children's Corner" Competitions.

**Notice to all Correspondents.**—N.B.—All letters referring to this page, which do not arrive at 38, Old Jewry, Cheapside, London, E.C., by the *first post on Thursdays*, and are not addressed PRIZE EDITOR, will in future be disqualified and disregarded.

No one will be permitted to win the Monthly Prizes twice in any one year, the Annual Prize being offered especially to prevent this.

